# Plasma neurofilament light chain as a biomarker of Alzheimer’s disease in Subjective Cognitive Decline and Mild Cognitive Impairment

**DOI:** 10.1007/s00415-022-11055-5

**Published:** 2022-03-14

**Authors:** Giulia Giacomucci, Salvatore Mazzeo, Silvia Bagnoli, Assunta Ingannato, Deborah Leccese, Valentina Berti, Sonia Padiglioni, Giulia Galdo, Camilla Ferrari, Sandro Sorbi, Valentina Bessi, Benedetta Nacmias

**Affiliations:** 1 Department of Neuroscience, Psychology, Drug Research and Child Health, University of Florence, Azienda Ospedaliero-Universitaria Careggi. Largo Brambilla, 3, 50134 Florence, Italy; 2 IRCCS Fondazione Don Carlo Gnocchi, Florence, Italy; 3 Department of Biomedical, Experimental and Clinical Sciences “Mario Serio”, University of Florence, Florence, Italy; 4 Nuclear Medicine Unit, Azienda Ospedaliero-Universitaria Careggi, Florence, Italy; 5 Regional Referral Centre for Relational Criticalities, Tuscany Region, Italy; 6 Unit Clinic of Organizations Careggi University Hospital, Florence, Italy

**Keywords:** Plasma neurofilament light chain, Subjective Cognitive Decline, Mild Cognitive Impairment, Alzheimer’s disease

## Abstract

**Introduction:**

Neurofilament light chain (NfL) is becoming increasingly notable in neurological diseases including AD, and it has been suggested as a new peripherical biomarker of neurodegeneration. We aimed to compare plasma NfL levels among Subjective Cognitive Decline (SCD), Mild Cognitive Impairment (MCI), and AD patients and to evaluate relationships between NfL and CSF biomarkers and neuropsychological scores.

**Materials and methods:**

We enrolled 110 patients (34 SCD, 53 MCI, and 23 AD), who underwent clinical and neuropsychological evaluation, *APOE* genotyping, and plasma NfL analysis. Ninety-one patients underwent at least one amyloid burden biomarker (CSF and/or amyloid PET); 86 patients also underwent CSF phosphorylated-tau (p-tau) and total-tau (t-tau) measurement. Patients were classified as A + if they presented at least one positive amyloid biomarker or A− if not.

**Results:**

NfL levels were significantly increased in AD and MCI compared to SCD patients. These differences depend on A status, e.g., SCD A + had lower NfLs than MCI A + but comparable with MCI A−. Similarly, MCI A + had higher NfL levels than MCI A−, but comparable with AD. NfL levels correlated with p-tau in SCD, with all CSF biomarkers in MCI patients. No correlations were found in AD subgroup. In SCD, NfL levels were negatively correlated with memory test scores.

**Conclusions:**

Plasma NfL levels might be a promising biomarker for neurodegeneration to discriminate cognitive decline due to AD from other conditions causing cognitive impairment in prodromal stages. Considering correlations with CSF p-tau and memory tests in SCD, NfL might be a useful peripheral biomarker also in preclinical phases of AD.

## Introduction

Alzheimer’s disease (AD) is a progressive debilitating neurodegenerative disease and the most prevalent type of dementia with an increasing incidence worldwide [1]. AD presents a presymptomatic period lasting from several years to decades [2]. Early stages of AD have been identified [3, 4]: Mild Cognitive Impairment (MCI) describes subjects with objective cognitive impairment without impact on instrumental activities of daily living and it is considered transitional between normal cognition and dementia [5]; Subjective Cognitive Decline (SCD) was defined as a self-experienced persistent decline in cognitive capacity in comparison with the subject’s previously normal status, during which the subject has normal age-, sex-, and education-adjusted performance on standardized cognitive tests [6]. SCD constitutes a heterogeneous group with many potential underlying causes and different trajectories [7]. Nevertheless, patients with SCD showed a higher incidence of progression to AD and higher prevalence of AD biomarkers as compared to individuals without [8, 9]. Therefore, the National Institute of Aging-Alzheimer’s Association (NIA-AA) included SCD as a first manifestation of the symptomatic stages of AD [10], followed by MCI [3].

The International Working Group (IWG) criteria [11] and NIA-AA criteria [3, 4, 12] proposed a new clinic-biological classification of AD, which was defined by its underlying pathologic processes that can be documented by post-mortem examination or in vivo by biomarkers. Consequently, the definition of AD shifted from a purely clinical to a biological construct. Moreover, since it has long been recognized that pathophysiological changes of AD begin many years before the development of clinical manifestations [13], NIA-AA research framework also proposed to group biomarkers into those of β amyloid deposition, pathologic tau, and neurodegeneration [AT(N)] to create different categories according to biomarkers positivity [14]. The ATN system described the “Alzheimer’s continuum”, starting from the biomarker evidence of Aβ deposition alone to AD, independently to clinical stage [14].

Current biomarkers of AD pathology are obtained through lumbar puncture (cerebrospinal fluid Aβ1-42, Aβ1-42/1–40, hyperphosphorylated tau and total tau) [15, 16] and positron emission tomography (PET) imaging (amyloid PET and tau PET) [17–19].

At the state of the art, investigation of AD biomarkers has reached a turning point. Research regarding possible disease-modifying therapies in AD has drawn attention toward the significance of early detection of AD [20]. Greatest challenge remains to identify new sensitive and specific biomarkers, obtainable with poorly invasive methods. These peripheral biomarkers might be useful in AD prodromal and preclinical phases, to allow a therapeutic intervention able to stop or, at least, slow down neurodegeneration.

Recently, neurofilament light chain (NfL) is becoming increasingly notable in a wide variety of neurological conditions including AD [21]. Belonging to the family of class IV intermediate filaments (IFs), NfL is a component of neuronal cytoskeleton, together with neurofilament heavy chain, neurofilament medium chain, and α-internexin in central nervous system and peripherin (class III IF) in peripheral nervous system [22]. Following neurodegeneration or axonal damage to both central and peripheral neurons, NfLs, can be released from neurons into the cerebrospinal fluid (CSF) and blood, playing as valuable marker of neuronal damage [23]. Recent studies have shown that NfL are increased in CSF and blood of AD patients: Mattsson et al. reported significantly higher plasma NfL levels in AD and MCI patients compared to controls [24], also describing an association with cognitive, biochemical, and imaging hallmarks of the disease. Hence, they proposed plasma NfL concentration as a noninvasive biomarker of AD. Moreover, recent studies on individuals who were carriers of AD mutations showed that blood NfL levels were already increased more than a decade before the estimated age of onset of clinical manifestations and the NfL peak rate of increase is observed near to the onset of symptoms [25–27]. However, only a few studies have analyzed the role of NfL plasma levels in SCD cohorts [28, 29].

We aim to assess quantitative differences in NfL plasma levels between SCD, MCI, and AD patients to investigate the role of NfL as a biomarker in the early stages of AD.

## Materials and methods

### Patients

Between April 2019 and July 2021, we consecutively collected 110 plasma samples from patients referred to the Centre for Alzheimer’s Disease and Adult Cognitive Disorders of Careggi Hospital in Florence. Blood samples were collected at the first evaluations for patients who come to our center for the first time, or at the check-up visit for patients who were regularly followed up at our center. Patients met the following inclusion criteria: (1) patients who received a clinical diagnosis of AD according to the NIA-AA criteria, including the atypical variant [12], (2) patients who received a clinical diagnosis of MCI according to NIA-AA criteria [3], and (3) patients who received a clinical diagnosis of SCD [6]. At the end, we included 34 SCD, 53 MCI, and 23 AD.

All patients underwent a comprehensive family and clinical history, neurological examination, extensive neuropsychological investigation, estimation of premorbid intelligence, and assessment of depression. Age at baseline corresponded to the age at the time of plasma collection. A positive family history was defined as one or more first-degree relatives with documented cognitive decline.

Eighty-six patients (23 SCD, 41 MCI, 22 AD) underwent CSF biomarker analysis (Aβ1-42, Aβ1-42/1–40 ratio, t-tau, p-tau). Twenty patients (6 SCD, 8 MCI, 6 AD) underwent cerebral amyloid PET from 1.11 years before and 1.66 years after the plasma collection (mean −0.03 ± 0.77). Ninety-one patients (24 SCD, 45 MCI, 22 AD) underwent at least one amyloid burden biomarkers (CSF and/or amyloid PET). Patients were classified as A + if at least one of the amyloid biomarkers (CSF or amyloid PET) revealed the presence of Aβ pathology and as A− if none of the biomarkers revealed the presence of Aβ pathology.

Correlations between CSF biomarkers and plasma NfL were performed in those patients who underwent CSF biomarkers analysis within a maximum of 2 years after or before NfL plasma sampling, including 83 patients (21 SCD, 40 MCI, and 21 AD) for these analyses.

One hundred and eight subjects (33 SCD, 52 MCI, 23 AD) underwent Apolipoprotein E (*APOE*) genotyping: *APOE* genotype was coded as *APOE* ε4- (no *APOE* ε4 alleles) and *APOE* ε4 + (presence of one or two *APOE* ε4 alleles).

Study procedures and data analysis were performed in accordance with the Declaration of Helsinki and with the ethical standards of the Committee on Human Experimentation of our Institute. The study was approved by the local Institutional Review Board (reference 15691oss). All individuals involved in this research agreed to participate and agreed to have details and results of the research about them published.

### Neuropsychological assessment

All subjects were evaluated by means of an extensive neuropsychological battery standardized and described in further detail elsewhere [30]. The battery consisted of global measurements (Mini-Mental State Examination), tasks exploring verbal and spatial short-term memory (Digit Span; Corsi Tapping Test), verbal long-term memory (Five Words and Paired Words Acquisition; Recall after 10 min; Recall after 24-h; Babcock Short Story Immediate and Delayed Recall), and language (Token Test; Category Fluency Task) [30]. Visual-spatial abilities were also evaluated by Rey–Osterrieth Complex Fig. copy and visuo-spatial long-term memory was assessed by means of recall of Rey–Osterrieth Complex Fig. test [31]; attention/executive function was explored by means of Dual Task [32], Phonemic Fluency Test [33], and Trail Making Test [34]. Everyday memory was assessed by means of Rivermead Behavioral Memory Test (RBMT) [35]. All raw test scores were adjusted for age, education, and gender according to the correction factor reported in validation studies for the Italian population [30–35].

To estimate the premorbid intelligence, all cases were assessed at baseline by the *Test di Intelligenza Breve* (TIB, i.e., Brief Intelligence Test) [36], an Italian version of the National Adult Reading Test (NART) [37]. The presence and severity of depressive symptoms was evaluated by means of the 22-item Hamilton Depression Rating Scale (HRSD) [38]. Cognitive complaints were explored at baseline using a survey based on the Memory Assessment Clinics-Questionnaire (MAC-Q) [39]. We defined the presence of cognitive complaints if participants perceived decline in cognitive capacity than in the past or if they reported difficulties in carrying out at least four of the following activities: remembering the name of a person just introduced to them; recalling telephone numbers or zip-codes used on a daily or weekly basis; recalling where they put objects in their home or office; remembering specific facts from a newspaper or magazine article just read; remembering the item(s) they intend to buy when they arrive at the grocery store or pharmacy.

For the purpose of the analysis, we considered the closest neuropsychological evaluation to NfLs sampling (mean time 0.03 ± 0.54 years [range −1.99; 2.43 years]).

### CSF collection and biomarkers analysis

The CSF samples were collected by lumbar puncture, then immediately centrifuged, and stored at − 80 °C until performing the analysis. Aβ1–42, Aβ42/40 ratio, t-tau, and p-tau were measured using a chemiluminescent enzyme immunoassay (CLEIA) analyzer LUMIPULSE G600 (Lumipulse Beta Amyloid1–40, Lumipulse Beta Amyloid1–42, Lumipulse GTotal Tau, and Lumipulse GPhospho Tau (181)). Cut-offs for normal values were: for Aβ1–42, > 670 pg/mL; Aβ42/40 ratio, > 0.062; t-tau, < 400 pg/mL; and p-tau, < 60 pg/mL [40]. Reagent kits were obtained from Fujirebio.

### Amyloid PET

Amyloid PET imaging was performed according to national and international standards [41], with any of the available fluorine18-labeled tracers (18Florbetaben [FBB]-Bayer-Pyramal, 18Flutemetamol [FMM]-General Electric). Images were rated as either positive or negative according to criteria defined by the manufacturers.

### Apolipoprotein E ε4 genotyping

A standard automated method (QIAcube, QIAGEN) was used to isolate DNA from peripheral blood samples. *APOE* genotypes were investigated by high-resolution melting analysis (HRMA) [42]. Two sets of PCR primers were designed to amplify the regions encompassing rs7412 [NC_000019.9:g[M13] [GG14] 0.45412079C > T] and rs429358 (NC_000019.9:g.45411941 T > C). The samples with known *APOE* genotypes, which had been validated by DNA sequencing, were used as standard references.

### Plasma neurofilament light chain analysis

Blood sample was collected and centrifuged within 2 h at 1300 rcf at 4 °C for 10 min and plasma was isolated and stored at −80 °C until tested. Plasma NfL analysis was performed with Simoa NF-Light SR-X kit (cat. No 103400) for human samples provided by Quanterix Corporation (Lexington, MA, USA) on the automatized Simoa SR-X platform (GBIO, Hangzhou, China), following the manufacturer’s instructions [43]. The Lower Limit of Quantification (LLOQ) and the Limit of Detection (LOD) provided by kit were 0.316 pg/mL and 0.0552 pg/mL, respectively. Plasma NfL concentration of all samples was detected in a single run basis. Quality controls, with a low NfL concentration of 5.08 pg/mL and with high NfL concentration of 169 pg/mL, were included in the array and tested with samples. A calibration curve was determined from measurements of serially diluted calibrators provided by Quanterix. Plasma samples and controls were diluted at a 1:4 ratio and measured in duplicate with calibrators.

### Statistical analysis

All statistical analysis were performed via IBM SPSS Statistics Software Version 25 (SPSS Inc., Chicago, USA) and the computing environment R 4.0.3 (R Foundation for Statistical Computing, Vienna, 2013). All *p* values were two-tailed and significance level for all analyses was set at α = 5%, corresponding to a threshold *p* of 0.05. Distribution of all variables was assessed through Shapiro–Wilk test. Patient groups were characterized by using means and standard deviations (SD), median and interquartile range (IQR), frequencies or percentages, and 95% confidence interval (95% CI) for continuous distributed variables, continuous non-normally distributed variables, and categorical variables, respectively. We used *t* test or non-parametric Mann–Whitney *U* tests for between groups’ comparisons, Pearson’s correlation coefficient or non-parametric Spearman’s ρ (rho) to evaluate correlations between groups’ numeric measures and Chi-square test to compare categorical data. We used two-sided Chi-square test to compare categorical data. We calculated the size effect by the Cohen’s *d* for normally distributed numeric measures, η^2^ for Mann–Whitney *U* test and the Cramer’s V for categorical data. Differences among groups in continuous variables were assessed through one-way ANOVA followed by Bonferroni post hoc test. We used a multiple regression to assess which variables independently influenced NfL levels.

## Results

### Demographic features

Demographic features are summarized in Table 1. Age at onset was significantly different among the three groups (*F* [2,100] = 16.61, *p* < 0.001), with a lower age of onset in SCD subjects (55.56 ± 7.52) as compared to MCI (64.75 ± 9.10, *p* < 0.001) and AD (66.79 ± 5.97, *p* < 0.001) patients. Age at baseline was significantly different among groups too (*F* [2,107] = 7.38, *p* = 0.001). SCD had higher education than AD patients (12.47 ± 3.46 vs 9.10 ± 3.97, *p* = 0.010). Mini-Mental State Examination (MMSE) was significantly different among the groups (*F* [2,91] = 55.94, *p* < 0.001) with poorer scores in AD (17.81 ± 7.45) compared to SCD (28.39 ± 1.52, *p* < 0.001) and MCI (27.51 ± 2.07, *p* < 0.001). Thirty-seven patients (34.25%) were *APOE* ε4 carriers: SCD were less frequently *APOE* ε4 carriers (18.18%) when compared to MCI (46.15%, χ^2^ 6.91, *p* = 0.009, Cramer’s V 0.285) and to AD (60.86%, χ^2^ 10.75, *p* = 0.001, Cramer’s V 0.438).

**Table 1:** Demographic features in Subjective Cognitive Decline (SCD), Mild Cognitive Impairment (MCI), and Alzheimer’s disease (AD) groups (110 patients)

	SCD	MCI	AD
	*N*° 34	*N*° 53	*N*° 23
Age at baseline in years	66.30 (± 8.09) *	72.78 (± 8.02) *	70.72 (± 6.08)
Age at onset in years	55.55 (± 7.52) ° ^ç^	64.75 (± 9.10) °	66.79 (± 5.97) ^ç^
Family history of AD	74.19%	60.00%	64.70%
Sex (M–F)	10–24	24–29	9–14
Years of education	12.47 (± 3.46) ^	11.31 (± 4.38)	9.10 (± 3.97) ^
MMSE	28.39 (± 1.52) ^ψ^	27.51 (± 2.07) ^ϖ^	17.81 (± 7.45) ^ψ ϖ^
*APOE* ɛ4 +	18.18% ^& §^	46.15% ^&^	60.86% ^§^
Plasma NfL (pg/ml)	13.19 (± 4.88) ^η ϒ^	22.32 (± 11.49) ^η^	24.54 (± 7.01) ^ϒ^
A +	37.50% [9/24] ^ϕ^	62.22% [28/45] ^μ^	100% [22/22] ^ϕ μ^

Values are reported as mean and standard deviation or frequencies or percentages for continuous variables and categorical variables, respectively. Statistically significantly different values between the groups are reported as underlined character. M: males; F: females; MMSE: Mini-Mental State Examination. The sample size for A + status is reported into brackets

^*^p = 0.001; °*p* < 0.001; ^ç^*p* < 0.001; ^*p* = 0.010; ^λ^; ^ψ^*p* < 0.001; ^ϖ^*p* < 0.001; ^&^χ^2^ 6.91, *p* = 0.009; ^§^χ^2^ 10.75, *p* = 0.001; ^η^*p* < 0.001; ^ϒ^*p* < 0.001; ^ϕ^ χ^2^ 20.40, *p* < 0.001; ^μ^ χ^2^ 11.13, *p* = 0.001

### Amyloidosis and neurodegeneration biomarkers analysis

Fifty-eight patients (67.44%) had at least one positive CSF amyloid biomarker (9 SCD, 27 MCI, 22 AD) (Table 2). Twelve patients (60.00%) (3 SCD, 5 MCI, 4 AD) had a positive cerebral amyloid PET. In summary, 91 patients (24 SCD, 45 MCI, 22 AD) underwent at least one biomarker (CSF and/or Amyloid PET). Based on the positivity for at least one cerebral amyloidosis biomarker, 59 (9 [37.5%] SCD, 28 [62.22%] MCI, 22 [100%] AD) were classified as A + . Percentage of A + patients were significantly different among groups, in particular between AD and MCI (χ^2^ 11.13, *p* = 0.001, Cramer’s V 0.408) and between AD and SCD (χ^2^ 20.40, *p* < 0.001, Cramer’s V 0.666).

**Table 2:** CSF biomarkers in Subjective Cognitive Decline (SCD), Mild Cognitive Impairment (MCI), and Alzheimer’s disease (AD) groups (86 patients)

	SCD	MCI	AD
	*N*° 23	*N*° 41	*N*° 22
Aβ1–42	1077.61 (± 410.34)*	888.10 (± 452.62)°	553.73 (± 127.82)*°
Aβ1–42/1–40	0.088 (± 0.028)^^ç^	0.065 (± 0.029)^	0.048 (± 0.011)^ç^
p-tau	68.57 (± 110.18)^λ^	78.59 (± 41.02)^ψ^	113.19 (± 67.79)^λ ψ^
t-tau	381.29 (± 227.90)^&^	510.14 (± 264.81)^§^	814.72 (± 375.32)^& §^

Values are reported as mean and standard deviation. Statistically significantly different values between the groups are reported as underlined character. *p < 0.001; °*p* = 0.004; ^*p* = 0.005; ^ç^*p* < 0.001; ^λ^*p* = 0.012; ^ψ^*p* = 0.018; ^&^*p* < 0.001; ^§^*p* < 0.001

### Comparisons of NfL levels among groups

To evaluate differences in NfL levels among SCD, MCI, and AD, one-way ANOVA with Bonferroni post hoc test was performed. NfL levels were significantly different among the three groups (*F* [2, 79] = 12.381, *p* < 0.001). At Bonferroni post hoc test, both MCI and AD had higher NfL levels as compared to SCD. In more details, NfL levels were significantly higher in AD (24.54 ± 7.01 pg/ml) and in MCI (22.32 ± 11.49 pg/ml) as compared to SCD patients (13.19 ± 4.88 pg/ml) (*p* < 0.001). NfL levels were not different between MCI and AD patients (*p* = 0.983) (Table 1).

**Fig. 1:**
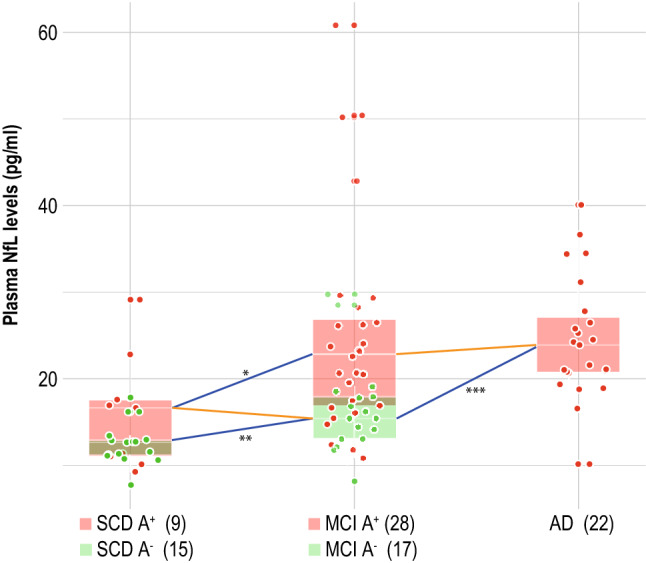
Plasma NfL levels in SCD, MCI, and AD patients, stratified by occurring of Aβ positivity

We compared NfL levels among SCD A + , SCD A−, MCI A + , MCI A−, and AD patients. NfL levels were significantly different between SCD A− and MCI A− (12.28 ± 2.36 vs 16.60 ± 5.47, *p* = 0.002, η^2^ = 0.31), between SCD A + and MCI A + (16.11 ± 6.60 vs 25.01 ± 12.27, *p* = 0.020, η^2^ = 0.14), between MCI A + and MCI A− (25.01 ± 12.27 vs 16.60 ± 5.47, *p* = 0.005, η^2^ = 0.18), and between AD and MCI A− patients (24.54 ± 7.01 vs 16.60 ± 5.47 *p* < 0.001, η^2^ = 0.37). No differences were detected between SCD A + and SCD A− (16.11 ± 6.60 vs 12.28 ± 2.36, *p* = 0.347, η^2^ = 0.04), SCD A + and MCI A– (*p* = 0.634, η^2^ = 0.01) and between MCI A + and AD (*p* = 0.455, η^2^ = 0.01) (Fig. 1).

### Association between NfL levels, demographic, cognitive, and genetic variables

**Fig. 2:**
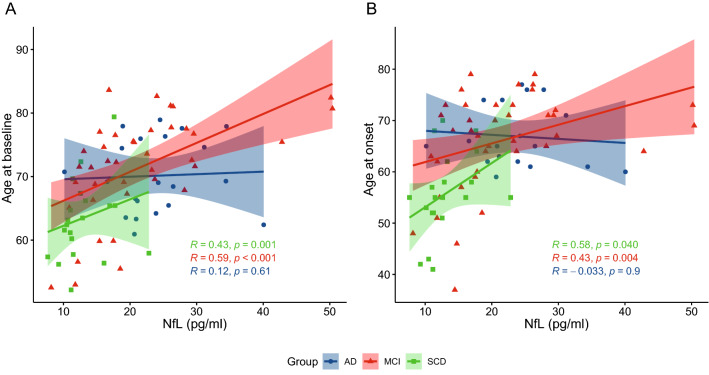
Correlation between plasma NfL levels, age at baseline, and age at onset of cognitive disturbs in SCD, MCI, and AD patients. Scatter plots with lines of best fit (95% CI) show the relationship between plasma NfL levels and age at baseline (**A**) and age at onset (**B**). NfL levels were significantly correlated with age at baseline and age at onset in SCD (Spearman’s ρ 0.529, *p* = 0.001 and Spearman’s ρ 0.364, *p* = 0.040, respectively) and MCI (Spearman’s ρ 0.550, *p* < 0.001 and Spearman’s ρ 0.416, *p* = 0.004, respectively) groups but not in AD

In the whole sample, NfL levels were significantly correlated with age at onset and with age at baseline in SCD (Spearman’s ρ 0.364, *p* = 0.040 and Spearman’s ρ 0.529, *p* = 0.001, respectively) and MCI (Spearman’s ρ 0.416, *p* = 0.004 and Spearman’s ρ 0.550, *p* < 0.001, respectively) groups but not in AD (Fig. 2). In the whole cohort, NfL levels were different between *APOE* ε4 + and ε4− (21.51 ± 9.31 vs 18.36 ± 9.23, *p* = 0.024, η^2^ = 0.05). No differences were detected between women and men.

Concerning neuropsychological measures, in SCD group, NfL levels were negatively correlated with memory tests (Rey auditory Verbal Learning test immediate recall RVLT-I, Spearman’s ρ = −0.616, *p* = 0.007; Rey auditory Verbal Learning test delayed recall RVLD, Spearman’s ρ = −0.767, *p* < 0.001; Babcock Short Story Delayed Recall, Spearman’s ρ = −0.467, *p* = 0.044). In MCI group, NfL levels were directly correlated with Spatial Span Forward (Spearman’s ρ = 0.605, *p* = 0.022). No correlations between NfL levels and neuropsychological tests were detected in AD patients.

### Correlations between NfL levels and CSF biomarkers

**Fig. 3:**
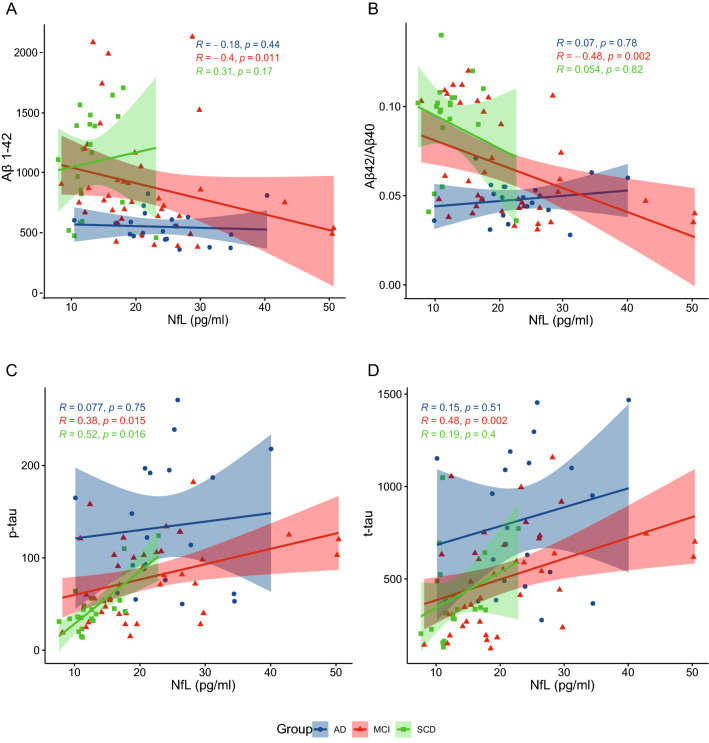
Correlation between plasma NfL levels and CSF biomarkers in SCD, MCI and AD patients. Scatter plots with lines of best fit (95% C.I.) show the relationship between NfL levels and Aβ1–42 (**A**), Aβ1–42/1–40 (**B**), p-tau (**C**), and t-tau (**D**)

In SCD patients, NfL levels were significantly correlated with p-tau (Spearman’s ρ = 0.518, *p* = 0.016), while correlations were found with Aβ1-42 (Spearman’s ρ = −0.399, *p* = 0.011), Aβ1-42/1–40 ratio (Spearman’s ρ = −0.478, *p* = 0.002), p-tau (Spearman’s ρ = 0.383, *p* = 0.015), and t-tau (Spearman’s ρ = 0.476, *p* = 0.002) in MCI group. No correlations with CSF biomarkers were detected in AD patients (Table 3) (Fig. 3).

**Table 3:** Correlations between plasma NfL levels and CSF biomarkers in Subjective Cognitive Decline (SCD), Mild Cognitive Impairment (MCI), and Alzheimer’s disease (AD) groups (83 patients)

	Plasma NfL levels					
	SCD *N*° 22		MCI *N*° 41		AD *N*° 22	
	Spearman’s ρ	*p*	Spearman’s ρ	*p*	Spearman’s ρ	*p*
Aβ1–42	0.309	0.174	**−0.399**	**0.011**	−0.177	0.444
Aβ1–42/1–40	0.054	0.816	−**0.498**	**0.002**	0.070	0.775
p-tau	**0.518**	**0.016**	**0.383**	**0.015**	0.077	0.748
t-tau	0.194	0.399	**0.476**	**0.002**	0.152	0.511

Significant differences are reported as bold character

### Multiple regression analysis

To analyze which factors might influence NfL levels, we ran a multiple regression analysis. We considered NfL levels as dependent variable, and diagnosis (SCD, MCI, or AD), age at baseline, CSF biomarker concentrations, years of education, sex, and *APOE* genotypes as covariates. The multiple regression model significantly predicted NfL levels (*F* [3, 73] = 251.74, *p* < 0.001, adj. *R*^2^ 0.899). Among the covariates, diagnosis (*B* = 2.897 [95% CI 0.389:5.406], *p* = 0.024), age at baseline (*B* = 0.282 [95% CI 0.179:0.385], *p* < 0.001), and Aβ1–42/1–40 ratio (*B* = −78.949 [95% CI −129.936: −27.962], *p* = 0.003) added statistically significantly to the prediction.

## Discussion

Our study aimed to explore differences in plasma NfL levels in SCD, MCI, and AD patients, focusing on both prodromal and preclinical stages of AD. As the main result, we found that plasma NfL levels were different among patients depending on the cognitive status (SCD, MCI, or AD) and on the underlying pathology (presence or absence of amyloid). In more details, MCI and AD patients had higher plasma NfL levels as compared to SCD. Furthermore, plasma NfL concentrations in MCI A + were higher than MCI A− and SCD A + , but consistent with AD patients.

These results could reflect the dynamic of neurodegeneration along the AD continuum, suggesting that NfL levels, starting from a limited neuronal death in SCD, increase until they reach a plateau. We might hypothesize that the peak of neural damage (mirrored by plasma NfL concentration) occurs during the transitional period from preclinical to prodromal stage, then reaching a plateau in dementia stage. Our results are in line with longitudinal studies on carriers of *APP*, *PSEN1*, and *PSEN2* mutations, showing that blood NfL levels increased more than a decade before the onset of clinical manifestations and the NfL peak was observed near to the onset of symptoms, suggesting an acceleration of neuronal death in the transitional zone between presymptomatic and symptomatic phases [25–27].

Furthermore, a previous study showed that plasma NfL levels were significantly higher in MCI and AD groups compared to controls. To the best of our knowledge, this is the first study analyzing plasma NfL levels in a well-defined SCD cohort. In fact, previous studies described volunteer subjects with normal cognition, not clearly diagnosed with SCD [29]. Mattson et al. described that plasma NfLs did not differ between Aβ-positive and Aβ-negative healthy controls, hypothesizing that any neuronal injury that may have occurred in Aβ-positive controls could be below the detection limit for plasma NfL [24]. Similarly, we did not find any difference between SCD A + and SCD A−. Nevertheless, NfL levels in SCD A + were lower than MCI A + patients, but consistent with NfL levels in MCI A−, while SCD A− had lower levels as compared to MCI A−. As a consequence, from a biological point of view, SCD A + patients are similar to patients with a greater cognitive decline (MCI). At the same way, MCI patients with at least one positive Aβ biomarkers had a neurodegenerative status consistent with patient diagnosed with AD. Interestingly, a recent study comparing CSF NfLs between SCD and cognitively healthy controls showed a difference between groups only in Aβ + individuals [44]. Taking together, this evidence leads to speculate that plasma NfL might precede the progression of cognitive decline in patients with Aβ pathology, being suitable as a prognostic biomarker in the AD continuum. Moreover, we also found that MCI A− patients had lower NfL levels as compared to MCI A + , but higher than SCD A−. This result may open an interesting research topic in the field of non-AD cognitive decline. Indeed, despite MCI A− patients do not belong to AD continuum, they represent a defined pathological entity driven by both degenerative and non-degenerative (including cerebrovascular, infective, metabolic, or psychiatric) conditions [45]. In particular, a study by Eratne et al. demonstrated the diagnostic utility of CSF NfL in differentiating neurodegenerative diseases from psychiatric disorders, with high accuracy [46]. Future studies may further investigate this point to assess the utility of NfLs in distinguishing psychiatric and neurodegenerative conditions among MCI not-due to AD.

Regarding the correlations between plasma NfL levels and CSF biomarkers, we found that NfL inversely correlated with Aβ1–42 and Aβ1–42/1–40 ratio and directly correlated with p-tau and t-tau in the MCI group, These results confirm the previous data [24] and may support the use of plasma NfL as a biomarker of AD-related biological changes in prodromal AD. In the SCD group, we found that NfLs directly correlated with p-tau. To the best of our knowledge, this is the first study analyzing plasmatic NfL levels and their relationship with CSF biomarkers in SCD population. This could furtherly support NfL as a potential biomarker of AD pathology also in preclinical phases, as p-tau is known to be a specific biomarker for AD [20, 47]. Interestingly, we found no correlations between NfL levels and CSF biomarkers in AD patients: we might speculate that this finding could be due to the fact that neurodegeneration reach a plateau, thus losing any correlation with AD typical biomarkers. The association between plasma NfL levels with AD is also supported by the correlations with neuropsychological measures. In SCD group, NfL levels were inversely correlated with scores at memory tests like RVLT-I, RVLT-D, and Babcock Short Story Delayed Recall. Similarly, Chatterjee et al. showed that plasma NfLs were inversely correlated with episodic memory, working memory, executive function, and the global composite score in cognitively normal elderly individuals [29].

This might be in contrast with the other literature data. Based on reports showing that NfLs were increased in many neurological diseases [48–50], NfLs have been considered as a non-specific biomarker for neural damage [24]. Our results might suggest that NfL accuracy might improve if we stratify patients according to the cognitive state. For this reason, we propose that further studies on biomarkers accuracy should be conducted separately in patients at different disease stages.

NfL levels were also directly correlated with age in SCD and MCI patients, as previously reported by other works [21, 24]. Our regression analysis showed that age, Aβ1–42/1–40 ratio, and the cognitive status (SCD, MCI, or AD) were all independently associated with NfL levels.

Our study presents some limitations: first, the relatively small number of patients, in particular in SCD subgroup. Second, as a healthy control group was not available, we could not verify if NfL levels in SCD were higher than NfL levels in individuals without SCD. Third, the design of this study is cross-sectional: it will be useful to conduct a longitudinal study to evaluate the rate of change in plasma NFLs over time and to assess the prognostic value of plasma NfL levels. Fourth, CSF biomarker analysis was not performed at the same time of plasma NfL sampling in all patients (considering a time range of maximum 2 years before or after): this aspect might influence our results as plasma NfLs kinetics are supposed to be nonlinear. Finally, the lack other degeneration biomarkers such as brain atrophy on MRI or brain hypometabolism on FDG-PET.

On the other hand, this study has some remarkable strengths: to the best of our knowledge, this is the first study which analyzed plasma NfLs and association with CSF biomarkers in well-characterized SCD patients, who met diagnostic criteria of SCD by Jessen et al. [6] and underwent CSF biomarkers analysis. Second, we applied a multidimensional approach, considering together clinical, cognitive, and biological variables.

In conclusion, our results support the hypothesis that plasmatic NfL levels change along the continuum of cognitive decline as a function of Aβ status. Moreover, this change might follow a nonlinear trend, with a peak and a stronger correlation with CSF biomarkers in the MCI phase. Therefore, NfLs might be useful peripheral biomarkers of neurodegeneration patients experiencing subjective or mild objective cognitive decline, to be integrated with neuropsychological and biological variables into a personalized medicine approach.
